# Supplementary material for: Deletion of wfs1 Impairs Oligodendrocyte Precursor Cells Dorsal Distribution and Myelination Through the wfs1-hmgcs1 Axis in Zebrafish
Source: Biology (Basel). 2026 Aug 16;15(16):1402. doi: 10.3390/biology15161402 (PMC13510151; doi:10.3390/biology15161402)
Supplement: Supplementary file 1 [file biology-15-01402-s001.zip › File S1-Transcriptome sequencing data and differentially expressed genes (DEGs) in wfs1-- zebrafish at 5 dpf/2.KEGG/wfs1KOvsWT/ALL/src/dre04114.html]

dre04114


- dre:100009657

- Up regulated genes

100009657\_1(1.89754653566672)

- dre:323883

- Down regulated genes

323883(-4.8491500403135)

- dre:323883

- Down regulated genes

323883(-4.8491500403135)

- dre:280649

- Up regulated genes

280649\_1(2.27023747569103)

- dre:192322

- Up regulated genes

336121(1.42904963424916)

- dre:565241

- Up regulated genes

565241(1.32977867526604)

- Down regulated genes

565241\_2(-6.09874532631292)

- dre:280649

- Up regulated genes

280649\_1(2.27023747569103)

- dre:323883

- Down regulated genes

323883(-4.8491500403135)

- dre:568726

- Up regulated genes

568726(1.14419230813088)
- dre:560807

- Up regulated genes

560807(1.71247301071773)

- dre:562552

- Up regulated genes

562552(1.50682290794657)

- dre:280649

- Up regulated genes

280649\_1(2.27023747569103)

- dre:797406

- Down regulated genes

797406\_2(-2.4994687612609)

- dre:797406

- Down regulated genes

797406\_2(-2.4994687612609)

- dre:562282

- Up regulated genes

562282(1.6446353235091)

- Down regulated genes

562282\_1(-5.61712483666314)

- dre:324475

- Down regulated genes

324475\_1(-3.125210470257)

- dre:100005148

- Up regulated genes

100005148\_1(1.71561398893188)

- dre:245702

- Up regulated genes

245702(1.27755220735994)

- Down regulated genes

245702\_1(-1.45433245048201)

- dre:324475

- Down regulated genes

324475\_1(-3.125210470257)

Close
